# Boosting the Clinical Translation of Organ-on-a-Chip Technology

**DOI:** 10.3390/bioengineering9100549

**Published:** 2022-10-14

**Authors:** David Caballero, Rui L. Reis, Subhas C. Kundu

**Affiliations:** 1 3B’s Research Group, I3Bs—Research Institute on Biomaterials, Biodegradables and Biomimetics, University of Minho, Headquarters of the European Institute of Excellence on Tissue Engineering and Regenerative Medicine, AvePark, Parque de Ciência e Tecnologia, Zona Industrial da Gandra, 4805-017 Barco, Portugal; 2 ICVS/3B’s—PT Government Associate Laboratory, 4704-553 Braga, Portugal

**Keywords:** organ-on-a-chip, microfluidics, drug screening, drug discovery, disease modelling, clinical translation, personalized medicine

## Abstract

Organ-on-a-chip devices have become a viable option for investigating critical physiological events and responses; this technology has matured substantially, and many systems have been reported for disease modeling or drug screening over the last decade. Despite the wide acceptance in the academic community, their adoption by clinical end-users is still a non-accomplished promise. The reasons behind this difficulty can be very diverse but most likely are related to the lack of predictive power, physiological relevance, and reliability necessary for being utilized in the clinical area. In this Perspective, we briefly discuss the main attributes of organ-on-a-chip platforms in academia and how these characteristics impede their easy translation to the clinic. We also discuss how academia, in conjunction with the industry, can contribute to boosting their adoption by proposing novel design concepts, fabrication methods, processes, and manufacturing materials, improving their standardization and versatility, and simplifying their manipulation and reusability.

## 1. Introduction

Undoubtedly, organ-on-a-chip (OoC) technology has revolutionized biomedical research by providing a biomimetic in vitro human-like habitat where cells and tissues can be studied in nearly native conditions with microfluidic flow [1]. As widely acknowledged, OoC platforms overcome several of the intrinsic limitations of the more widely-established pre-clinical in vitro and in vivo models, and therefore, are considered an attractive tool for clinical experimentation [2,3,4]. Since the invention of the pioneering lung-on-a-chip by the Ingber laboratory [5], a myriad of OoC models of tissues and their pathologies have been reported [2,6,7,8]. Despite the key insights obtained in academic research regarding cell, tissue, and organ functions, disease pathophysiology, and drug efficacy, the promised expectations of OoC in clinics are far from being accomplished. Indeed, OoC was proposed as a revolutionary technology to eliminate animal experimentation [9]. However, several researchers and end-users consider that OoC technology might have already reached its exploitation plateau without achieving the initial expectations, as the existing gap between academic and clinical work is still challenging to bridge [10,11]. Its future utility will therefore depend on their capacity for translating this disruptive technology to the clinical area and their ability to help physicians in decision-making. 

The basic concept of OoC technology has not significantly progressed since the initial prototypes introduced by academic laboratories. Most of these devices are commonly manufactured using polydimethylsiloxane (PDMS) elastomer and conventional microfabrication techniques (UV- and soft-lithography). The tendency of PDMS-based chips to absorb small hydrophobic molecules (which may jeopardize drug experiments), the high lab-to-lab variability, and the low throughput limit the clinical translation of OoC in its current state [12]. Much attention has now been focused on evolving OoC into more relevant and standardized systems using designs and materials with improved physiological relevance with the promise of broadening their adoption and translational applications [10]. In this regard, several OoC companies have emerged and developed microfluidic systems displaying generic and versatile designs, eliminating the need to use complex instrumentation, thus making OoC assays more standardized, simpler and at a reduced cost [2]. However, besides the availability of these commercial devices, this technology is still significantly costly, and complex compared to traditional preclinical, experimental tools. More importantly, it is still considered a relatively time-consuming and complex technology to work with due to its reduced dimensions, which makes chip manipulation not easy, and the need to integrate other instrumentation associated with microfluidics, such as tubing, connectors, or pumps. Nevertheless, OoC are certainly cheaper, less time-consuming, and simpler to implement in clinics than animal models. For a significant number of biomedical researchers, the traditional conception of organ-on-a-chip systems will remain unaltered, and only minor changes may apply, mainly related to the biological or biomaterial part. However, if this technology is intended to be adopted by the end-users, particularly clinicians and the pharma/biotech industry, the OoC paradigm may need a certain degree of rethinking to make it simpler, reliable, affordable, and fast. 

In this Perspective, we briefly discuss the current paradigm of OoC technology and its main pitfall. Particularly, we focus on how this technology born in academic laboratories may need to be adapted for bringing it to the next level and attract the interest of non-specialized end-users, particularly clinicians, who are traditionally reluctant to this unconventional technology, but aiming to explore novel, but well-proven, tools to address biomedical problems. Finally, this manuscript also seeks to generate a space of debate by providing novel ideas, design concepts, and applications for designing the future route of OoC technology.

## 2. Organs-On-A-Chip: Rethinking the Canonical Vision of Tissue/Organ Modelling

OoC have maturated substantially during the last decade and a large plethora of in vitro platforms have already been developed [1,2,10,13]. The canonical concept of OoC, defined as “microfluidic systems containing human cells that recapitulate the minimal functional unit of a human organ or tissue in an engineered microphysiological environment,” has been expanded towards a much broader concept and applications during the last years. In this regard, not only specific tissues and organs have been reproduced on-chip with relatively good biomimicry, but complex (multi-) tissue functions and responses have also been described [14]. Indeed, some OoC have reached a high degree of sophistication with a diversity of chips displaying very specific designs, constructions, and implementations, which allows them to faithfully recapitulate the in vivo physiology, predict human response, and provide quantitative data [4,15,16,17]. However, the different involved scientific communities (academic, industry, and clinic) do not always share the same interests, and the design, manufacturing methods, applications, and functions of OoC have to be adapted to satisfy the needs of each of them. 

### 2.1. OoC in Academic Research

The contribution of OoC to fundamental biological sciences is undeniable. Many pathophysiological processes have been identified and recapitulated using microfluidic platforms that closely mimic the native scenario. Particularly, dynamic physiological events, such as fluid flow, gradient generation, or mechanical stimulation, can be integrated within the cell-laden chip, reaching an unprecedented level of biomimicry [18]; this complexity is, *per se,* appropriate for understanding the mechanistic determinants of pathologies, the mechanism of action of drugs, or unmasking unexpected toxicological issues. However, this technical and biological sophistication is univocally associated with significant difficulties generally incompatible with the needed efficiency or productivity typically required by the industry and clinics.

Biomedical researchers use reverse engineering to build biomimetic OoC models integrating the critical building blocks of human tissues to reproduce their functional working units [19]; this strategy allows the production of sophisticated chips with accurate architectures that mimic the in vivo scenario. Next, a well-controlled amount of cells, proteins, or other molecules are injected through the microfabricated channels and reservoirs under controllable culture and physiological flow conditions. Cell activity and response to injected drugs or compounds can be continuously monitored using conventional microscopy techniques; this approach to model a single entity can be extended to multiple tissues or organs, resulting in multi-organ-on-a-chip (multi-OoC) devices [14]. For this, scaling ratios between the different embedded tissues (i.e., size, number of cells, metabolic activity, flow, etc.) must be considered to maintain physiological relevance, crucial for predicting in vivo events, such as drug screening [20]. 

Traditionally, commercial cell lines are employed in academic research for building OoC models; these cells can be an excellent option due to their robustness, simplicity of culture and manipulation, avoiding the harsh culturing protocols of primary cells. During the last years, though, it has been widely accepted that commercial cell lines provide limited information about physiological processes and drug responses, with high lab-to-lab variability, and lack of predictive power that hamper their clinical applicability [21]. More physiologically-relevant cells, such as induced pluripotent stem cells (iPSCs) or primary cells from specific donors, can be employed to develop more realistic models of healthy and diseased conditions; these cells are much more delicate to handle (specific culture and media are required for maintaining their native phenotypes) and difficult to obtain from the donor (e.g., neural cells). Additionally, primary cells should be continuously analyzed to evaluate their correct phenotype and have to be employed at low passages. For iPSCs, well-established protocols for generating isogenic cell lines have been reported, particularly for OoC applications [22]. However, immature phenotypes are usually obtained for specific tissues, which may limit the relevancy of the obtained data [23]. The inclusion of environmental signals and physical stimuli on-chip can contribute to a better (and faster) mimicking of adult tissue. The development of organoids from these cells for emulating the functional characteristics of the in vivo tissue and their integration within the microfluidic platforms as organoids-on-a-chip can open new avenues in biomedical research due to their well-reported physiological relevance [24]. Importantly, all the developed OoC platforms, independently of their building blocks and biological material, must be experimentally validated by comparing the gathered information with robust (i.e., validated) in vitro and in vivo models to check their performance in terms of accuracy and predictive power [13]. For this, using validated cell sources and culture media, animal models, and the analysis of biomarkers of proven clinical relevance may boost confidence in this technology to bridge the gap between academic labs and clinics.

Despite the high degree of sophistication and the intrinsic complexity of OoC models reported in the literature, OoC experimentation in academic research is univocally linked to reductionism, not only in human biology but also in the supporting extracellular matrix. Traditionally, cells have been encapsulated within natural-derived extracellular matrix protein-based hydrogels, mainly from animal origin, such as Matrigel^TM^, collagen, or gelatin (or blends of them) [25]; this type of non-human materials can trigger undesired immune responses or transmit diseases, impacting data quality [26]. Currently, xeno-free human-derived materials, such as platelet lysate, or the fabrication of decellularized or bioengineered cell-derived matrices using human material as a source, are preferred due to their enhanced biocompatibility [27]. Indeed, several works have employed human-derived materials within OoC platforms to boost their clinical relevance, some of them fully-defined in composition [28,29,30,31]. Particularly, human-based matrices provide multiple advantages compared to traditional biomaterials, such as a native-like composition or the ability to recapitulate the fibrillary architecture and mechanical properties of the in vivo cell habitat [32]. A particular feature of these matrices is their remarkable ability to recover after being completely dehydrated; this can be very attractive for commercialization purposes, where the microfluidic device can be pre-integrated with a lyophilized matrix, which can be reconstituted prior to starting the assay at the clinical laboratory; this approach could decrease the complexity, time and cost of traditional OoC experiments. 

Another natural biomaterial for encapsulating cells within OoC is silk fibroin [33]. Silk fibroin has excellent biocompatibility, low immunogenicity, robustness, oxygen permeability, and tunable mechanical properties, being FDA-approved for specific medical applications [27]. Hydrogels made of silk fibroin can therefore be a good and cheap option as supporting ECM materials replacing more conventional materials. Finally, an alternative to natural-derived materials is synthetic hydrogels (e.g., poly (ethylene glycol), synthetic peptides, etc.), which offer good control over the mechanical properties of hydrogels and do not display the traditional batch-to-batch variability and uncontrollable degradation rate of their natural counterparts. However, most synthetic hydrogels do not provide cell-adhesive molecules and cannot be degraded by cells. Therefore, this could jeopardize the study of long-term dynamic processes in which cells migrate across the hydrogel (e.g., cancer cell invasion), thus losing the required biomimicry. Even though they can be chemically modified to address these drawbacks, they have attracted less attention in the OoC academic community, which still prefers using traditional natural-derived matrices with well-proven properties, thus avoiding unnecessary functionalizations.

Finally, the canonical vision of OoC based on microfabricated chips has been challenged by novel, cutting-edge manufacturing and analytical technologies; this has resulted in the development of more advanced microphysiological systems that better model, manipulate, and monitor cell activity. For example, miniaturized sensors and actuators have been integrated within OoC platforms to provide quantitative and real-time information about the physiology of the system while allowing the remote manipulation of the biological material (see Section 2.3), being a significant step toward the clinical translation of OoC [34]. Notably, 3D (bio) printing has been explored to generate chip architectures and dimensions not achievable with traditional microfabrication techniques, and, therefore, can recapitulate the complexity of the in vivo scenario in a more satisfactory manner [35]. However, this and other approaches described herein only permit the fabrication of a relatively low number of chips per day, and, therefore, are not sufficient for the high throughput needs of the clinic.

### 2.2. OoC in Industry

The industrialization of OoC technology has significantly contributed to expanding its throughput, analytical, and quantitation capabilities; this has been a continuous demand from the pharmaceutical and biotechnological industry aiming for new and better screening methods to overcome the high attrition rates in drug discovery and preclinical evaluation [36]. Due to the superior ability to reproduce human physiology, OoC can provide the satisfactory performance that traditional screening platforms, such as Petri dishes, static 3D culture platforms, or animal models, are incapable of, particularly for mimicking the dynamic physiology (i.e., fluid flow) and compartmentalized micro-architecture of human tissues and organs [2,37]. Some pharmaceutical and biotechnological companies have realized the enormous potential of OoC for parallelizing experiments [38,39,40], which could improve the success rate in drug discovery and reduce the astonishing attrition rates and associated costs [41]. For this reason, the high-throughput characteristics of OoC initially developed by academic researchers have been improved by integrating them into 96-well laboratory plates. Importantly, these plates are compatible with other standard analytical techniques/instruments (e.g., plate readers), thus increasing the amount of data that can be extracted from a single experiment [42]; this has contributed to decreasing the cost and duration of the assay, which is a strict requirement for the industry and the clinical end-users. Some of these well-plates have been engineered with arrays of microelectrode-containing biosensors to continuously monitor the presence (or absence) of critical prognostic biomarkers. Indeed, the use of biochemical/physical biosensors within microfluidic devices has attracted a lot of attention from the scientific community, where a vast amount of literature is available [43]. However, as recently described by López-Muñoz et al., limited advances in the integration on-chip of self-operative sensing devices and their validation (i.e., good analytical performance) in real settings have been achieved [44]; this might be a consequence of the non-adequate functionalization of the sensing electrodes that threatens the reliability of the assay. In this regard, robust sensor functionalization and regeneration methods would be convenient to extend the lifetime of the sensing device, increase the reliability of the device, and decrease the associated costs of using new chips. Alternatively, incorporating stimuli-responsive microgels cleaved in the presence of specific compounds could provide a more efficient alternative to traditional electrochemical assays [45].

Overall, the synergistic combination of microfluidics and biosensors can revolutionize data collection for pharmaceutical and biotechnological industries by providing real-time data about the efficacy and side-effects of drugs in a rapid and throughput manner, thus optimizing the pipeline of drug discovery and screening. To achieve this, the typical microfabrication techniques used by academic labs are not appropriate; they have been replaced by the industry with more efficient manufacturing processes, such as injection molding or additive manufacturing tools for the rapid and large-scale production of OoC [46,47]. Similarly, chip designs and operating methods have been standardized to decrease manufacturing costs and, importantly, to permit the direct comparison of the obtained results between different laboratories, including clinical pharmacological analysis at hospitals, aiming to decrease the traditional variability reported in OoC assays in academic research. In some cases, such rigid designs are not convenient for cell experimentation, and more flexibility is required while maintaining a certain degree of standardization. The use of pre-fabricated universal microfluidic modules, each of them containing different living tissues (e.g., liver, cardiac, tumor, and others), has been reported as a solution to provide this capability that is needed in specific industrial settings to speed up the screening of candidate molecules [48]; this modular microfluidics configuration enables the pre-culturing of the different tissue/organ modules using distinct conditions before their simple plug & play assembling. The interconnection of tissue modules shows excellent promise for modelling complex multi-tissue interactions, particularly for assessing the altered pharmacokinetics/pharmacodynamics of drugs and their metabolites; this would enable the so-called ADMET (administration, distribution, metabolism, excretion, and toxicity) profiling of drugs [49,50]. However, the higher the number of interconnected modules/tissues, the higher the technical and biological complexity, which, univocally, may affect the viability of the cultured cells. Therefore, there is a need to simplify the complexity and provide accessible solutions for clinical in vitro applications [51]. Finally, translational capacity is also challenged by using more sophisticated systems, mainly due to the need for a common—universal—culture media.

Finally, for industrial applications, conventional pumping mechanisms, such as pressure-driven apparatus (e.g., syringe pumps), are not convenient due to the difficulty in connecting the tubes and the possibility of introducing bubbles, thus not meeting the industrial standards of efficiency. For this reason, and with the end-users in mind, some industrial manufacturers introduced since the very beginning gravity-driven systems in their OoC assays; this simpler alternative can significantly reduce the complexity, manipulation, and associated cost of the experiment, enabling the assay to be performed in any location and within tiny areas (e.g., micro-incubators). A few disadvantages of this approach may include waste accumulation, particularly for long-term experiments, and uncontrollable—bi-directional—flow rates, as reported elsewhere [13]. Large reservoirs can be engineered in the inlets/outlets for the former to keep the media in good condition. For the latter, automatic liquid handling systems, such as robotic pipetting tools, may be employed, providing the speed and high-throughput characteristics needed by the industry and requested by the clinics. 

### 2.3. OoC for Clinicians

The substantial level of robustness and standardization provided by industrial manufacturers might favor the adoption of OoC by clinicians in the near future to improve the predictability of therapeutics in patients [18]. Indeed, clinicians were identified very early on as the targeted end-users of OoC technology for assessing the efficacy of drugs before testing them on patients or for identifying novel therapeutic targets without the need to use (humanized) animal models [52,53]. However, the current level of complexity/development of OoC makes this vision still challenging, even though promising steps are being taken toward this direction. One impeding parameter is the long culture times needed to build a biomimetic microenvironment on-chip, typically in the range of days to weeks. Despite the demonstrated physiological relevance of iPSCs, primary cells or patient-derived organoids in clinical decision-making [30], their long culture time within perfusable chips might be incompatible with specific pathologies, such as in cancer, where the diagnosis and therapeutic decisions must be initiated quickly to improve the prognosis of patients. For clinical applications, it is more practical to use tissue biopsies gathered from patients encapsulated in single-route chips to evaluate their short- and long-term response to therapeutic compounds. Alternatively, purchasing pre-established preclinical OoC models of physiological and pathological processes could significantly facilitate and accelerate all the steps involved in pre-clinical experimentation, thus eliminating the challenge of establishing the OoC platforms in the lab. Importantly, these pre-established models are already commercially-available by well-established OoC companies, such as Emulate^TM^ or MIMETAS^TM^, and have been validated in static and dynamic flow conditions. In this regard, the use of flow may be questionable depending on the intended application. In some cases, the flow of fresh cell culture medium may be preferred to maintain cell culture in pristine conditions or analyze specific biomarkers released by the tissue. However, it has been shown that media exchange can impact other critical physiological processes, such as cellular proliferation, due to the perturbation of endogenous levels of growth factors [54]; these perturbations can be continuously monitored by integrating miniaturized biosensors on-chip (see Section 2.2); this specific feature of OoC systems is undoubtedly precious for clinical applications for collecting a large amount of data. For example, on-chip biosensors can enable the in situ detection/quantification of predictive/prognostic biomarkers and their distribution or the measurement of physiological parameters (e.g., pH, O_2_, H_2_O_2_, etc.) in the cellular compartment or bodily fluids. However, it is worth emphasizing that the measurement of physiological parameters on-chip must be trustful and already clinically validated [55,56].

Next, the material utilized for chip fabrication and cell culture is an essential factor to be considered for developing OoC for clinical applications [57]. Single-use, FDA-approved, and sterile materials are undoubtedly the best choice regardless of PDMS, which shows serious problems, particularly a high absorbance for small hydrophobic molecules, as mentioned earlier [12]. The use of traditional polystyrene material already validated for the culture of tissues and massively employed in cell biology is a valid option for the massive fabrication of OoC platforms for clinical applications. Further, polystyrene has a high hydrophobicity that avoids the deposition/adhesion of molecules and proteins in the walls. For biological material, patient-derived biopsies may be utilized on-chip to generate miniaturized patient avatars to predict how a drug will work for a specific person [58]; this will enable the evaluation of new treatments in a low-cost, personalized, and rapid manner [59]. However, a single biopsy cannot account for the screening of a large battery of drugs; this limitation can be surpassed by dissecting the tissue into multiple microscopic pieces that retain the appropriate cellular environment; this idea was recently explored using micro-droplets massively generated using a microfluidic device and containing tissue cell mixtures [60]. The miniaturized tissues within the droplets retained key physiological features of the native material (e.g., histopathological morphology, differentiation capacity, and genetic expression), and therefore had the potential for being applied in realistic drug testing on-chip. 

Finally, much has been discussed in the literature on using OoC for personalized medicine in clinics [2], where the efficacy of drugs (or combinations of several therapeutic compounds) and dose regimes can be rapidly tested on-chip using a patient biopsy. For this, automation is essential to perform all this set of tests rapidly. As discussed above, some microfluidic chips have already been specifically designed for being employed in microplate formats for high-content screening assays, improving the speed of data collection and with the promise of improving medical decisions [61]. Despite the intense efforts toward this direction and the number of possibilities that can be achieved with OoC (see ref. [10] for a detailed review), the clinical expectations generated by OoC by the scientific community (it was ranked 6th among the top ten emerging technologies in 2016 by the World Economic Forum [52]) should be down-toned to avoid a general discouragement on this technology. It is imperative to strengthen the—long-lasting—collaborations between academic labs, industrial partners, and clinicians to boost its clinical potential and increase the adoption rates. Despite still being a minority, some of these communities have established R&D collaborations to evaluate the toxicity of drugs in OoC [58,62]. In this process, strict adaptations will need to be done where relevant regulatory agencies have to play a fundamental role in ensuring that this medical technology meets international regulatory requirements for generating trustful, reproducible and robust products [63]. Notably, it should be followed the official “guidance document on the validation and international acceptance of new or updated test methods for hazard assessment” developed by the Organization for Economic Co-operation and Development [64], and comply with the current regulations when placing new medical devices on the market (In Europe, Directive 93/42/EEC for medical devices and Directive 98/79/EC for in vitro diagnostic devices; in the US, 21 CFR Part 58 guidelines and FD&C Act Section 507 program by the FDA) and the ISO 9001:2015 by the International Organization of Standardization [65] to catapult this technology. 

## 3. Discussion

Recent advances in nanotechnology and tissue engineering have led to the development of miniaturized physiologically-relevant biological modelling and testing systems with the potential to revolutionize biomedical research, drug discovery, and future clinical decisions. In this regard, OoC has attracted much attention not only from the academic community but also from industrial stakeholders due to its improved predictive power and performance compared to traditional 2D/3D testing platforms and animal models that fail to predict the outcome of a drug in humans [2]. Clinicians, the intended end-users of this technology, are generally still not entirely convinced about the promised capabilities of OoC for predicting therapeutic response and faithfully modelling patient physiology. In academic research and in an increasing number of industrial settings, OoC has become an established alternative to conventional screening methods. Unquestionably, OoC developed in academic research has provided numerous outstanding results, but certain drawbacks might threaten their clinical acceptance. To promote its adoption by the clinic as a regular screening tool, academic and industrial developers must adapt this technology to clinical practitioners to perform more efficient, simpler, reliable, robust and rapid diagnostic/screening tests, enhancing their compatibility with current medical technologies [66]. Indeed, significant efforts have been invested in improving the physiological relevance of OoC (e.g., new designs—modular or standardized architectures; integration of human-based materials; high throughput characteristics; simpler manipulation; improved biology; and others), obtaining key cell biology insights. The adaptation of OoC to future, or growing technologies, such as miniaturized analytical sensors, artificial intelligence and machine learning systems for automated biomarker detection, image processing and data analysis, would provide unprecedented advantages to the clinical team in decision-making [67]. Next, even though the future of OoC for clinical applications is optimistic, the challenges mentioned above, mainly related to OoC validation, standardization, throughput, and compatibility with existing analytic/imaging technologies, will need to be addressed. Similarly, the lack of endocrine and immune responses of OoC will need to be carefully managed; this will result in OoC systems that are easier to use, more predictive, and more automatable [13].

Finally, it is worth mentioning a fundamental aspect typically overlooked in tissue modelling: circadian rhythms; this is of utmost importance, particularly for long-term experimentation, and therefore, might be fundamental for specific clinical applications; these natural periodic processes affect biological material, such as cells [68]. As such, they must also be integrated within OoC experimentation to improve the prediction of the obtained responses, such as those related to the susceptibility of drugs, since circadian rhythms impact those responses. The integration of circadian rhythms on-chip has been typically neglected, and very few works have focused on them, such as investigating the oscillatory nature of metabolic signals and showcasing the importance of temporal fluctuations in tissue physiology [69]. 

**Figure 1:**
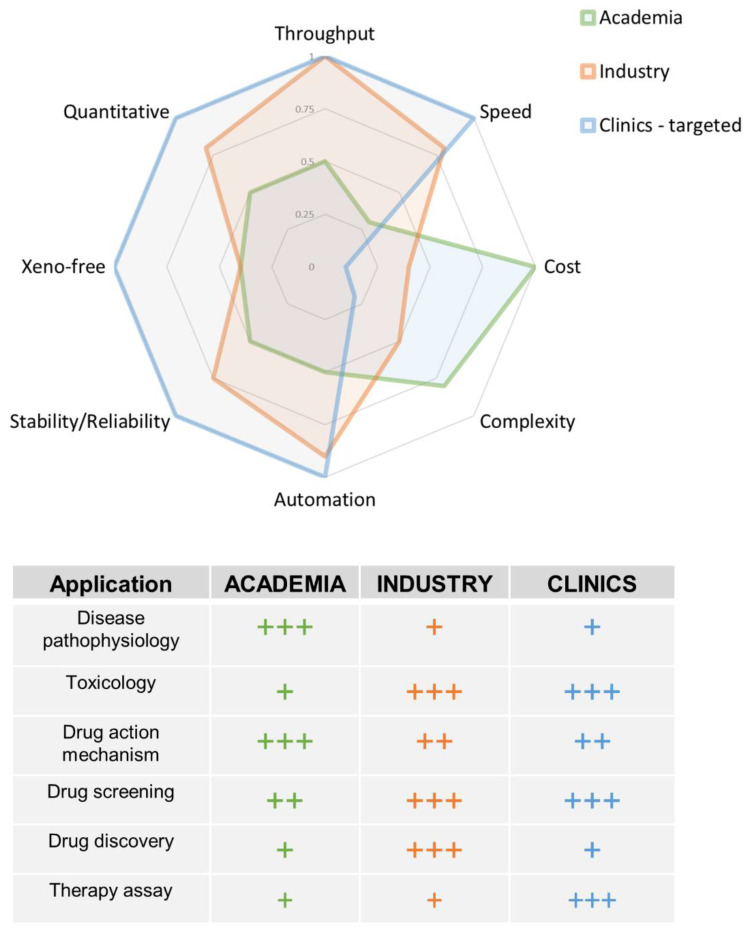
Boosting OoC into the clinics. (**Top**) Radar scheme comparing the main typical features and (**bottom**) applications of OoC for academia (green) and industry (orange), and the desired characteristics for being applied in the clinic (blue). The scores in the radar diagram range from 0 to 1, representing 1 the ideal performance. The symbols “+” in the table highlight the degree of importance of each application in academia, industry and clinic, representing “+++” the highest value.

Altogether, it is expected to see outstanding developments in OoC systems in clinics during the next decade. In this regard, Figure 1 summarizes the main applications and features (technical and biological) of OoC in academia and industry, and the desired characteristics they should display to boost their translation into the clinics. The listed applications and characteristic features are not exhaustive, and many others may be missing. However, it may serve as a practical guide to better understand the needs and interests of each community working in OoC [5,46,54,60]; this will result in developing novel therapeutic compounds released in the market and optimizing therapies, thus univocally improving patient prognosis. 

## 4. Conclusions

Organ-on-a-chip technology has an enormous potential to improve our understanding of the molecular mechanisms involved in the onset of human diseases by modelling tissue/organ function within a microfluidic chip. OoC can bridge the existing gap between obsolete flat cell culture assays, non-predictive animal testing, and complex human experimentation. As such, OoC can significantly ameliorate the high attrition rates of newly-discovered drugs by providing a rapid, high-throughput, low-cost, and predictive technology while implementing the 3Rs (replacement, reduction, and refinement) directive on animal experimentation. Despite the impressive expansion of OoC technology during the last decade, its clinical adoption has evolved at a different pace due to the technical complexity of a technology that emerged from academic labs, which has faced difficulties adapting to the medical community. To bridge this gap, simpler, more standardized, robust, reproducible, automated and reliable platforms must be manufactured by the industrial stakeholders with the collaboration of the other communities; this does not mean that OoC need to be down-graded in terms of biological complexity and physiological relevance, but they have to be adapted to the respective communities with distinct needs. If intended to be accepted by clinicians, OoC must be validated in an operation environment. That is, they should show robust reproducibility in terms of analytical performance with clinically-relevant samples/cells and a high ability to predict drug response on encapsulated tissues.

## Data Availability

Not applicable.
